# Genetic aetiology of primary adrenal insufficiency in Chinese children

**DOI:** 10.1186/s12920-021-01021-x

**Published:** 2021-06-30

**Authors:** Zhuo Chang, Wei Lu, Zhuhui Zhao, Li Xi, Xiaojing Li, Rong Ye, Jinwen Ni, Zhou Pei, Miaoying Zhang, Ruoqian Cheng, Zhangqian Zheng, Chengjun Sun, Jing Wu, Feihong Luo

**Affiliations:** Department of Pediatric Endocrinology and Inherited Metabolic Diseases, Children’s Hospital of Fudan University, 399 Wan Yuan Road, Shanghai, 201102 People’s Republic of China

**Keywords:** Primary adrenal insufficiency, Genetic diagnosis, Whole-exome sequencing, Congenital adrenal hyperplasia, Triple A syndrome, X-linked adrenoleukodystrophy

## Abstract

**Background:**

Primary adrenal insufficiency (PAI) is life-threatening, and a definitive aetiological diagnosis is essential for management and prognostication. We conducted this study to investigate the genetic aetiologies of PAI in South China and explore their clinical features.

**Methods:**

Seventy children were enrolled in this cross-sectional study. Clinical information was collected, and combined genetic tests were performed according to the children’s manifestations. Statistical analysis was performed among the different groups. In silico or in vitro experiments were applied to determine the pathogenicity of novel variants.

**Results:**

Among the 70 children, 84.3% (59/70) were diagnosed with congenital adrenal hyperplasia (CAH), and 21-hydroxylase deficiency (21-OHD) was genetically confirmed in 91.5% of these cases. Salt wasting (SW), simple virilization (SV), and non-classic (NC) CAH accounted for 66.1% (39/59), 30.5% (18/59), and 3.4% (2/59) of the cases, respectively. The 17-hydroxyprogesterone (17-OHP) and testosterone (TES) levels were significantly higher in children with SW than with SV. The 17-OHP and cortisol levels in female SW patients were significantly higher than those in males. The 17-OHP, cortisol, dehydroepiandrosterone (DHEAS) and TES levels in female SW patients were significantly higher than those in female SV patients. Additionally, 72.7% (8/11) of uncharacterized PAI patients had positive genetic findings. Among all the patients, two novel variants in the *CYP21A2* gene (c.833dupT and c.651 + 2T > G) were found. A microdeletion (Xp21.2–21.3) and five novel variants, including 2 in the *NR0B1* gene (c.323–324CG > GA and c.1231_1234delCTCA), 2 in the *AAAS* gene (c.399 + 1G > A and c.250delT) and 1 in the *NNT* gene (c.2274delT), were detected. The novel variant c.399 + 1G > A in the *AAAS* gene was further confirmed to lead to exon 4 skipping during mRNA transcription and produce a truncated ALADIN protein.

**Conclusions:**

We found ethnicity-based differences in the *CYP21A2* gene variant spectrum among different study populations. Female 21-OHD patients tended to have higher 17-OHP and TES levels, which warrants caution in relation to the effects of virilization. Novel gene variants detected in the *CYP21A2*, *NR0B1*, *AAAS* and *NNT* genes expanded the genetic spectrum of PAI, however, further improvement of genetic testing tools beyond our protocol are still needed to uncover the complete aetiology of PAI in children.

**Supplementary Information:**

The online version contains supplementary material available at 10.1186/s12920-021-01021-x.

## Background

Primary adrenal insufficiency (PAI) is an infrequent but critical clinical condition caused by inadequate secretion of steroid hormones, mainly cortisol and/or aldosterone, as well as adrenal sex steroids [1]. PAI in children is very different from that in adults, as congenital disorders caused by genetic aberrations are much more frequent [2]. Based on the affected biological process, the aetiology of inherited PAI can be categorized into several groups [3]. The first is impaired steroidogenesis, such as congenital lipoid adrenal hyperplasia caused by mutations in *STAR* [4] and 21-hydroxylase deficiency caused by *CYP21A2* [5]. This group includes different abnormalities in the steps of steroid biosynthesis. Congenital adrenal hyperplasia (CAH), which is caused by 21-hydroxylase deficiency, accounts for most paediatric PAI cases, ranging from approximately 70–80% of cases [2, 3]. The second group is adrenal hypoplasia, including X-linked adrenal hypoplasia congenita (AHC) caused by *NR0B1* [6], adrenal hypoplasia caused by steroidogenic factor-1/*NR5A1* [6], IMAGe syndrome caused by *CDKN1C* [7] and MIRAGE syndrome caused by *SAMD9* [8]. Patients in this group usually lack all adrenocortical hormones, and some of them may have accompanying symptoms such as intrauterine growth restriction and genital abnormalities. The third is adrenocorticotrophic hormone (ACTH) resistance, including familial glucocorticoid deficiency type 1 caused by *MC2R* [9] and familial glucocorticoid deficiency type 2 caused by *MRAP* [10]. Children in this group usually have a preserved renin-aldosterone axis, and plasma ACTH is often markedly increased despite normal glucocorticoid treatment. Adrenal destruction constitutes another group, consisting of Allgrove syndrome caused by *AAAS* [11], nicotinamide nucleotide transhydrogenase deficiency caused by *NNT* [12]and thioredoxin reductase deficiency caused by *TXNRD2* [13]. The next group is complex lipid metabolism, including X-linked adrenoleukodystrophy (X-ALD) caused by *ABCD1* [14], neonatal adrenoleukodystrophy caused by *PEX1* [15] and Wolman disease caused by *LIPA* [16]. The last group is autoimmune destruction, including isolated autoimmune adrenalitis associated with *CLTA-4, HLA-DR3, HLA-DR4*, *HLA-B8* and *BACH2* [3].

Among the remaining patients, the aetiology varies greatly based on the study population and test platform. In Asia, Amano et al. [17] applied target gene sequencing and discovered that when excluding 21-hydroxylase deficiency and 3β-hydroxysteroid dehydrogenase deficiency, genetic defects were detected in 84.7% of patients, with *STAR* (32.2%), *NR0B1* (30.5%) and *SAMD9* (11.9%) accounting for the top three. In North America, Tsai et al. [18] used the same method to determine the cause in 81.8% of patients, and the most common was *STAR* (45.5%). In the Middle East, Guran et al. [19] employed a gene panel plus next-generation sequencing and found 81% positive genetic diagnoses, with *MC2R* (26.3%), *NR0B1* (12.6%) and *STAR* (11.6%) as the three most common causes. In Europe, Chan et al. [20] applied whole exome sequencing (WES) and made genetic diagnoses in 41.9% of patients, with *CYP11A1* (14.0%), *NR0B1* (9.3%) and *AAAS* (4.7%) as the most affected genes.

In clinical practice, the differential diagnosis and management of paediatric PAI are quite challenging [21]. Eyal et al. reviewed the epidemiology and risk factors for adrenal crises in children with adrenal insufficiency (AI) between 1990 and 2017 at four Israeli paediatric endocrinology units and found that diagnosis and long-term management of paediatric patients remained a challenge [22]. Adrenal crises are life-threatening emergencies, but studies on the rate and risk factors for adrenal crises in children with AI are scarce [22]. Currently, the exact cause of more than 5% of paediatric PAI cases is undetermined despite continual detection of novel genetic causes [23]. Considering the limited data for Chinese PAI children, we used multiple molecular testing strategies to explore genetic causes of PAI in Chinese children and to determine the correlation between genotype and phenotype.

## Subjects and method

### Subjects

Seventy children were diagnosed with PAI from July 2012 to August 2017 at Children’s Hospital of Fudan University according to published criteria [24]: (i) initial symptoms suggesting low cortisol levels (e.g., hyperpigmentation, fatigue, failure to thrive, vomiting, electrolyte disturbances); (ii) laboratory examinations indicating high plasma ACTH levels accompanied by low or normal cortisol levels and a less than 500 nmol/L peak during the ACTH stimulation test; and (iii) availability for genetic testing. All the patients received detailed clinical evaluations, and all female CAH patients received Prader genital scale (PGS) scores for external genitalia. Patients with hyperkalaemia, hyponatremia, elevated serum 17-hydroxyprogesterone (17-OHP), and external genitalia virilization manifestations were initially suspected of having 21-hydroxylase deficiency (21-OHD), which was further verified through *CYP21A2* gene testing [25, 26]. The diagnostic algorithm for PAI patients is shown in Additional file 1: Fig. S1. The study was approved by the Ethics Committee of the Children’s Hospital of Fudan University. Written informed consent for the study was obtained from all patients’ parents.

### Biochemical measurements

Venous blood samples were drawn at approximately 8 am in the morning, and all hormones were tested within 2 h, including serum ACTH (IMMULITE 2000 ACTH, Siemens, UK), cortisol (Access Cortisol, Beckman Coulter, USA), dehydroepiandrosterone sulphate (DHEA-S; Access DHEA-S, Beckman Coulter), testosterone (TES; Access Testosterone, Beckman Coulter) and 17-OHP (17 Alpha-hydroxyprogesterone Radioimmunoassay Kit, Cisbio Bioassays, France).

### Molecular analysis

Genomic DNA was extracted from peripheral lymphocytes, and four genetic tests were performed according to individual clinical presentations: (i) for 59 patients who presented with cortisol deficiency, androgen excess and/or elevated 17-OHP, the *CYP21A2* gene was amplified using PCR (Additional file 4: Table S1) and double checked using multiplex ligation-dependent probe amplification (MLPA; SALSA MLPA Probemix, MRC-Holland, Netherlands); (ii) for 2 patients with developmental delay/intellectual disability or congenital anomalies, array-based comparative genomic hybridization (Array-CGH, Agilent Technologies, CA, USA) was performed; and (iii) the remaining patients underwent WES (SureSelectXT Human All Exon Kit V6, Agilent Technologies, CA, USA).

Variants found in all genetic tests except for Array-CGH were verified by Sanger sequencing in the probands and some of the parents. Truncating variants such as nonsense, frameshift, and splice site variants, gene deletions and previously reported variants were regarded as pathogenic variants. For novel variants with frequencies lower than 0.01 in the ExAC (http://exac.broadinstitute.org/), dbSNP147 (http://www.ncbi.nlm.nih.gov/SNP/) and 1000 Genomes (http://www.internationalgenome.org) databases, pathogenicity was predicted with SIFT (http://sift.jcvi.org), PolyPhen-2 (http://genetics.bwh.harvard.edu/pph2) and Mutation Taster (http://www.mutationtaster.org).

### Determination of the *AAAS* mRNA structure with the c.399 + 1G > A variant

One patient harboured a mutant *AAAS* gene with c.399 + 1G > A. Subsequent mRNA analysis was performed to test its pathogenicity. Human Splicing Finder (HSF) 3.0 software (http://www.umd.be/HSF3/index.html) was used to predict the potential donor or acceptor site alteration of the altered splice site. Total RNA was isolated from the peripheral blood of the proband, the parents and normal control individuals (Total RNA Kit, TIANGEN BIOTECH, Beijing, China). The forward primer 5’-TGGATCAATCTTCCTGTCCTACAAC-3’, covering the first 25 bp of exon 2, and the reverse primer 5’-TACAGTGAACAGCAGTCGG-3, 46 bp upstream of the end of exon 11, were used to amplify the transcripts of both the wild-type and mutant *AAAS* genes. The amplification product of normal individuals was predicted to be 919 bp. The PCR products were separated by agarose electrophoresis and then extracted from the agarose gel (TIANgel Midi Purification Kit, TIANGEN BIOTECH, Beijing, China). The diversity of transcripts was verified by Sanger sequencing.

### Genotype–phenotype correlation in children with CAH

CAH patient genotypes were categorized into 5 groups as previously described [27, 28]. Briefly, group 0 included null variants on both alleles; group A included homozygotes for the I2G variant or compound heterozygotes for the I2G variant and a group 0 variant; group B included patients who were either homozygous or compound heterozygous for the p.I173N variant with group 0 or A variants; group C included patients who were either homozygous or compound heterozygous for the p.P31L or p.V281L variants with group 0, A or B variants; and group D included unidentified variants and new variants. The genotype–phenotype correlation was assessed in the patients with both genetic and initial clinical information. The positive predictive value (PPV) was calculated as the percentage of patients with the predicted phenotype. The predicted phenotypes were salt wasting (SW) for groups 0 and A, simple virilization (SV) for group B, and non-classic (NC) for group C.

### Statistical analysis

SPSS Statistics (19.0) software was used for statistical analysis. The data are expressed as the median and inter quartile range (IQR) due to non-normal distributions. Comparisons between groups were performed using the Mann–Whitney U test without the missing data, and differences were regarded as significant if p < 0.05.

## Results

### Clinical characteristics of children with CAH

**Fig. 1:**
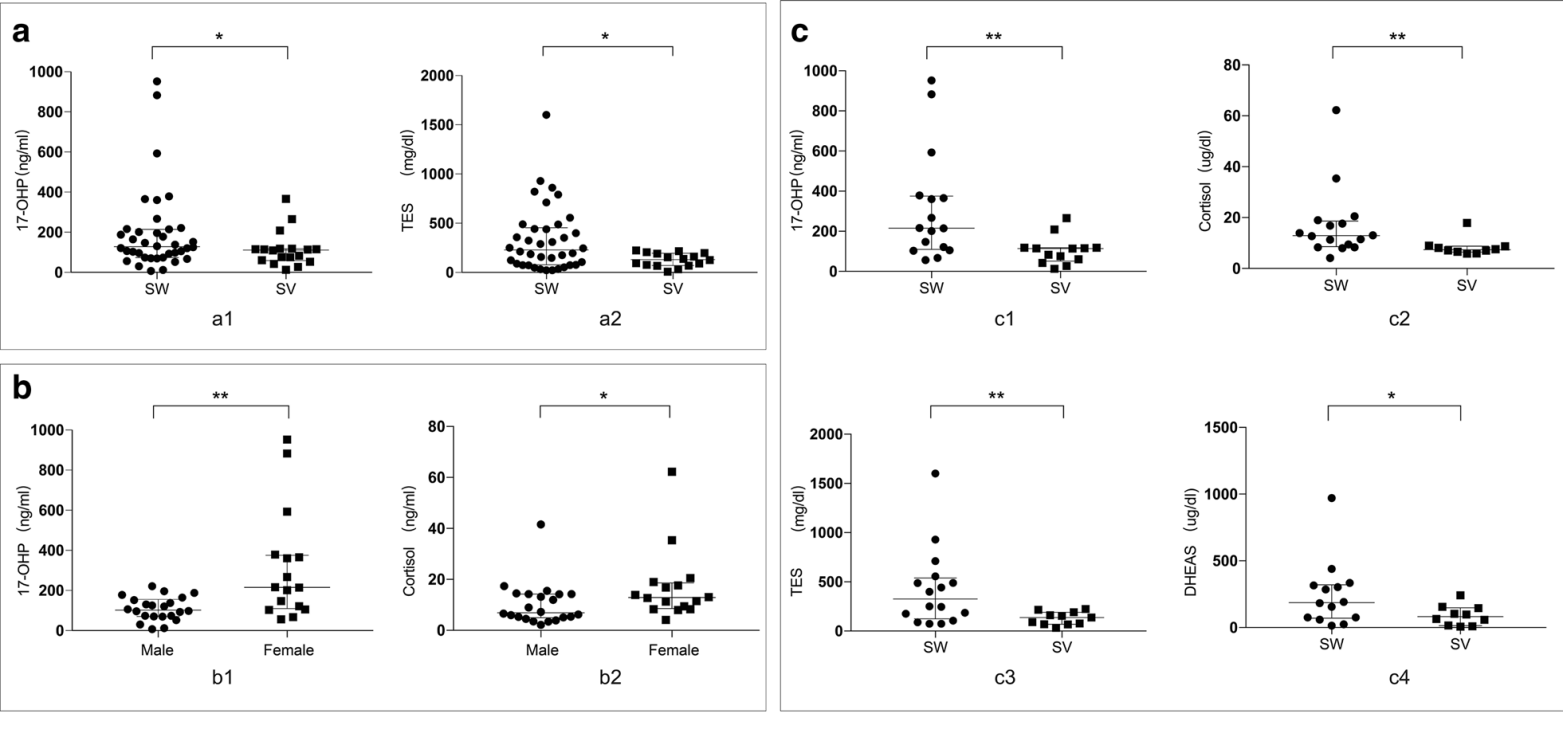
Differences in relevant serum hormones in CAH patients. The data are expressed as the medians (IQRs) due to non-normal distributions. Comparisons between groups were performed using the Mann–Whitney U test. The 17-OHP level and the serum TES level were significantly higher in SW patients than in SV patients [**a1**: SW 127.93 ng/ml (141.08 ng/ml), n = 38; SV 96.21 ng/ml (78.00 ng/ml), n = 18; p = 0.018. **a2**: SW 228.90 ng/dl (376.90 ng/dl), n = 38; SV 131.64 ng/dl (125.51 ng/dl), n = 16; p = 0.034]. In SW patients, females had significantly higher serum 17-OHP levels [**b1**: female 215.36 ng/ml (265.48 ng/ml), n = 16; male 101.93 ng/ml (84.81 ng/ml), n = 22; p = 0.003] and cortisol levels [**b2**: female 12.88 µg/dl (10.05 µg/dl), n = 16; male 6.88 µg/dl (9.27 µg/dl) n = 22; p = 0.033]. In female children, the SW group had increased 17-OHP [**c1**: SW 215.36 ng/ml (265.48 ng/ml), n = 16; SV 113.63 ng/ml (83.39 ng/ml), n = 13; p = 0.004], cortisol [**c2:** SW 12.88 µg/dl (10.05 µg/dl), n = 16; SV 7.35 µg/dl (2.37 µg/dl), n = 10; p = 0.005], TES [**c3**: SW 325.40 ng/dl (415.44 ng/dl), n = 16; SV 138.27 ng/dl (122.43 ng/dl), n = 11; p = 0.008] and DHEAS [**c4**: SW 187.45 µg/dl (248.88 µg/dl), n = 14; SV 81.35 µg/dl (133.63 µg/dl), n = 10; p = 0.042] levels. The initial serum levels of 17-OHP in one SW male patient, cortisol in three SV female patients, TES in one SW male patient and two SV female patients, and DHEAS in two SW female patients and three SV female patients were not included due to inconsistent testing methods. Hormone normal ranges ACTH < 46 pg/ml, cortisol 5–25 µg/dl, 17-OHP1 month-1 year 1.06–40.41 ng/ml, 1 year-13 years 0.07–1.53 ng/ml, TES female 0–31 ng/dl, male 0–6 years 3–32 ng/dl, 7–12 years 3–68 ng/dl, DHEAS 35–430 µg/dl

Among the 70 children, 84.3% (59/70) had CAH, and 15.7% (11/70) had uncharacterized PAI. The sex ratio was almost equal among CAH patients (male 29, female 30), and a male predominance (81.8%, 9/11) was found for uncharacterized PAI, as 4 of the male patients had X-linked conditions. Among the 59 CAH patients, SW, SV and NC patients accounted for 66.1% (39/59), 30.5% (18/59), and 3.4% (2/59) of the samples, respectively. A PGS score ≥ 3 was found in 93.8% (15/16) of female with SW and 38.5% (5/13) of female with SV (Additional file 5: Table S2). TES and 17-OHP levels were significantly higher in children with SW than in children with SV (p = 0.018 and p = 0.034, respectively). Among SW patients, 17-OHP and cortisol levels in females were significantly higher than those in males (p = 0.003 and p = 0.033, respectively).Among female patents, the 17-OHP, cortisol, dehydroepiandrosterone (DHEAS) and TES levels in SW patients were significantly higher than those in SV patients (p = 0.004, p = 0.005, p = 0.042, p = 0.008, respectively) (Fig. 1).

### Mutation spectra in the *CYP21A2* gene and the genotype–phenotype correlation in 21-OHD patients

Combined Sanger sequencing and MLPA tests yielded 91.5% positive (54/59) variant findings. The most common variants were c.293-13C > G (31.3%), Del (18.6%), p.I173N (17.0%), E3 Δ8 (5.1%), p.R483PfsX58 (4.2%) and p.R357W (3.4%) (Table 1). Two novel variants, namely, c.833dupT (NC_000006.1: g.32007876dupT; NM_000500.9: c.833dupT, reference protein NP_000491: p.279GfsX17) and c.651 + 2T > G (NC_000006.1: g.32007486T > G; NM_000500.9, c.651 + 2T > G, reference protein NP_000491: p.?), were found in two patients (Additional file 2: Fig. S2). The variant c.833dupT in exon 7 of the *CYP21A2* gene was found in an SV patient with the p.I173N variant on the other allele. The splicing variant c.651 + 2T > G at the end of exon 5 of the *CYP21A2* gene was found in an SW patient with the c.293-13C > G variant on the other allele (Table 2). The protein was predicted to be p.(184D_217Rdel). Complete concordance was observed only in group 0, while the concordance values were 83.3% and 84.6% for groups A and B, respectively. Two SW patients in group D with only one pathogenic allele presented with elevated 17-OHP, growth retardation, hyperkalaemia, hyponatremia and adrenal hyperplasia. The other two SW patients in group D with the same presentations had no pathogenic variants in the *CYP21A2* gene. All the above patients refused to undergo further genetic examination. One NC patient in group D who developed increased 17-OHP and dehydroepiandrosterone with mild pigmentation and bilateral adrenal hyperplasia had a monoallelic variant (Additional file 6: Table S3).

**Table 1:** Spectrum of *CYP21A2* variants in Asian patients and in other ethnic groups

Country	Sample	c.293-13C > G (%)	Del (%)	I173N (%)	G111_113VfsX4 (%)	R483PfsX58 (%)	R357W (%)	P31L (%)	Q319X (%)	E6 cluster (%)	V282L (%)	Other (%)
Croatia [27]	93	34.9	18.8	11.3	2.2	0.0	16.7	5.9	4.8	2.2	0.0	3.2
Brazil [37]	480	21.1	9.0	7.5	1.8	0.0	5.4	0.6	6.1	1.2	26.6	20.7
Serbia [42]	61	18.5	13.0	2.8	1.4	0.0	11.1	13.0	4.6	0.9	4.6	30.1
Argentina [35]	454	20.6	11.2	8.2	0.8	1.5	4.2	0.7	6.7	2.0	26.2	19.7
UK [41]	153	30.3	45.0	7.0	0.0	0.0	16.7	0.0	4.8	0.0	0.0	3.8
Tunisia [36]	50	6.0	22.0	8.0	2.0	0.0	1.0	1.0	26.0	1.0	12.0	9.0
Germany + Austria [50]	538	29.2	29.6	13.1	2.5	0.0	4.1	2.6	4.6	1.5	7.8	5.0
China [33]	43	20.9	8.6	36.0	1.2	0.0	2.3	7.0	7.0	2.3	4.7	10.0
China [32]	230	35.0	19.6	14.3	4.3	0.0	5.9	0.2	4.6	1.3	0.2	14.6
China [31]	30	38.3	15.0	11.7	0.0	0.0	5.0	1.7	1.7	3.3	0.0	23.3
China [30]	35	27.0	27.0	17.6	1.4	0.0	9.5	0.0	5.4	1.4	1.4	9.3
China [34]	166	42.5	23.8	12.7	3.0	2.1	6.0	0.0	5.1	0.9	0.0	3.9
China (this study)	59	31.4	18.6	17.0	5.1	4.2	3.4	1.7	0.9	0.9	0.9	16.1

**Table 2:** Genotype–phenotype correlations in CAH patients

Group	Allele 1	Allele 2	Phenotype			Positive predictive value for the predicted phenotype (%)
			SW	SV	NC	
Group 0	Del	Del	5	0	0	100%
	Del	R483PfsX58	1	0	0	
	Del	R357W	1	0	0	
	Del	L308FfsX5-Q319X-R357W	1	0	0	
	Del	P31L- c.293-13C > G- E3 Δ8	1	0	0	
	E3 Δ8-Q319X	c.293-13C > G- Q319X	1	0	0	
	Q319X	L308FfsX5- Q319X	1	0	0	
	R357W	E3 Δ8	1	0	0	
Group A	c.293-13C > G	c.293-13C > G	6	2	0	83.3%
	c.293-13C > G	Del	5	0	0	
	c.293-13C > G	E3 Δ8	2	1	0	
	c.293-13C > G	R357W	2	0	0	
	c.293-13C > G	R483PfsX58	2	0	0	
	c.293-13C > G	E6 cluster	1	0	0	
	c.293-13C > G	c.293-13C > G- E3 Δ8	1	0	0	
	c.293-13C > G- E3 Δ8	G292S	0	1	0	
	c.293-13C > G	c.1223-1G > A	1	0	0	
Group B	I173N	I173N	0	5	0	84.6%
	I173N	Del	0	2	0	
	I173N	c.293-13C > G	1	1	0	
	I173N	E3 Δ8	0	2	0	
	I173N	R483PfsX58	0	1	0	
	I173N	L308FfsX6	1	0	0	
Group C	V282L	I173N	0	0	1	Not applicable^a^
	P31L	Del	0	1	0	
	P31L	L308FfsX6	0	1	0	
Group D	p.279GfsX17	I173N	0	1	0	Not applicable^b^
	c.651 + 2 T > G	c.293-13C > G	1	0	0	
	V305M	No pathologic mutations detected	0	0	1	
	c.293-13C > G	No pathologic mutations detected	1	0	0	
	R483PfsX58	No pathologic mutations detected	1	0	0	
	No pathologic mutations detected	No pathologic mutations detected	2	0	0	
Total	39	18	2			

*Del* large fragment deletion, *E3 Δ8* c.331_339delGAGACTAC, *E6 cluster* c. [7010T > A;713T > A;719T > A], p. [I237N; V238E; M240K]

^a^The positive predictive value for the expected phenotype was not calculated due to the small sample size

^b^The positive predictive value for the expected phenotype was not calculated because] enzyme activity was unable to predict novel mutations

### Mutation spectra and the genotype–phenotype correlation in children with uncharacterized PAI

Highly diverse genetic defects were detected in 72.7% (8/11) of probands from 7 families, and five novel variants were detected in five patients, including 2 in the *NR0B1* gene (NC_000023.1: g.30327156-30327157CG > GA; NM_000475.5, c.323–324 CG > GA, reference protein NP_000466: p.S108X and NC_000023.1: g.30322873-30322876delCTCA; NM_000475.5, c.1231_1234delCTCA, reference protein NP_000466:p.L411Vfs*6), 2 in the *AAAS* gene (NC_000012.1: g.53709118G > A; NM_015665.6, c.399 + 1G > A, reference protein NP_056480: p.? and NC_000012.1: g.53714350delT; NM_015665.6: c.250delT, reference protein NP_056480: p.W84Gfs*10) and 1 in the *NNT* gene (NC_000005.1: g.43656054delT; NM_182977.3, c.2274delT, reference protein NP_036475: p.I758Mfs*10) (Table 3, Additional file 3: Fig. S3). Case 1 (mutant *ABCD1* gene) showed elevated very long-chain fatty acid levels. Case 2 (*IL1RAP-NR0B1-GK* deletion, Xp21.2–21.3) presented with undetectable adrenal glands, increased triglyceride levels, hypothyroidism and cryptorchidism, in addition to typical AI and mineralocorticoid deficiency. Among the four patients with reduced adrenal gland size, 75% (3/4; cases 2, 3, and 4) harboured *NR0B1* gene pathogenic variants. Cases 5, 6 and 7 (mutant *AAAS* gene) had alacrima with or without achalasia and/or neurological symptoms and were all offspring of consanguineous parents. Case 8 (mutant *NNT* gene) presented no other features in addition to PAI (Table 3).

**Table 3:** Clinical features of uncharacterized PAI patients

Proband (Sex)	Age (years)	Gene variants	Electrolyte				Adrenal CT imaging	Main clinical presentation
			ACTH (pg/ml)	Cortisol (baseline /peak) (µg/dl)	17-OHP (ng/ml)	K^+^/ Na^+^ (mmol/l)		
Case 1 (Male)	5.2	*ABCD1* NC_000023.1: g.153005609; NM_000033.4: c.1552 C > T, reference protein NP_000024: p.R518W (rs128624224) X-link	> 1250	1.46/1.58	0.16	4.1/140	Normal	pigmentation, alacrima, epilepsy, consanguineous parents
Case 2 (Male)	0.1	*IL1RAP-NR0B1-GK* Microdeletion of Xp21.2–21.3(2.6 Mb) Hom	> 1250	4.83/ND	3.37	6.2/130	Undetectable	Pigmentation, mineralocorticoid deficiency, adrenal crisis, abnormal liver function, elevated triglyceride, hypothyroidism, cryptorchidism
Case 3 (Male)	3.7	*NR0B1* NC_000023.1: g.30327157–30,327,156 CG > GA; NM_000475.5:c.323–324 CG > GA, reference protein NP_000466: p. S108X X-link, novel	> 1250	4.00/4.09	0.06	5.4/122	Undetectable	Pigmentation, mineralocorticoid deficiency, adrenal crisis, abdominal lymphadenectasis
Case 4 (Male)	9.8	*NR0B1* NC_000023.1: g.30322873–30,322,876 delCTCA; NM_000475.5: c.1231_1234delCTCA, reference protein NP_000466: p.L411Vfs*6 X-link, de novel	> 1250	1.75/2.17	0.16	5.6/125	Tiny adrenal glands	Pigmentation, mineralocorticoid deficiency
Case 5 (Male)	8.4	*AAAS* NC_000012.1: g.53709118G > A; NM_015665.6: c.399 + 1G > A, reference protein: NP_056480: p.? Hom, novel	710.00	0.15/ND	0.01	4.2/144	Normal	Pigmentation, alacrima, achalasia, consanguineous parents
Case 6 (Female)	5.5	*AAAS* NC_000012.1: g.53714350delT; NM_015665.6: c.250delT, reference protein NP_056480: p.W84Gfs*10 Hom, novel	> 1250	1.00/ND	0.03	4.4/142	Normal	Pigmentation, alacrima, epilepsy, consanguineous parents
Case 7 (Female)	3.2	*AAAS* NC_000012.1; g.53714350delT; NM_015665.6, c.250delT, reference protein NP_056480: p.W84Gfs*10 Hom, novel	> 1250	1.00/ND	0.01	3.4/135	Normal	Pigmentation, alacrima, consanguineous parents
Case 8 (Male)	1.9	*NNT* NC_000005.1: g.43656054delT; NM_182977.3:c.2274delT, reference protein NP_036475: p.I758Mfs*10 Hom, novel	482.1	0.27/ND	0.03	5.3/136.3	Normal	Pigmentation
Case 9 (Male)	0.2	–	313	2.42/3.22	0.19	4.5/134	Normal	Pigmentation, low hypophysis
Case 10 (Male)	5.0	–	241	9.21/11.10	0.11	4.0/141	Tiny adrenal glands	Pigmentation, microphallus, short stature, microcephaly, hepatomegaly, splenomegaly
Case 11 (Male)	2.2	–	250	14.64/16.53	0.40	3.9/134	Normal	Pigmentation

*17-OHP* 17-hydroxyprogesterone, *ACTH* adrenocorticotrophic hormone, *TES* testosterone, *DHEAS* dehydroepiandrosterone, *ND* not done. Normal ranges ACTH < 46 pg/ml, cortisol 5–25 µg/dl, 17-OHP1 month-1 year 1.06–40.41 ng/ml, 1–13 years 0.07–1.53 ng/ml, TES female 0–31 ng/dl, male 0–6 years 3–32 ng/dl, 7–12 years 3–68 ng/dl, DHEAS 35–430 µg/dl

### Pathogenicity analysis of the novel splicing variant c.399 + 1G > A

**Fig. 2:**
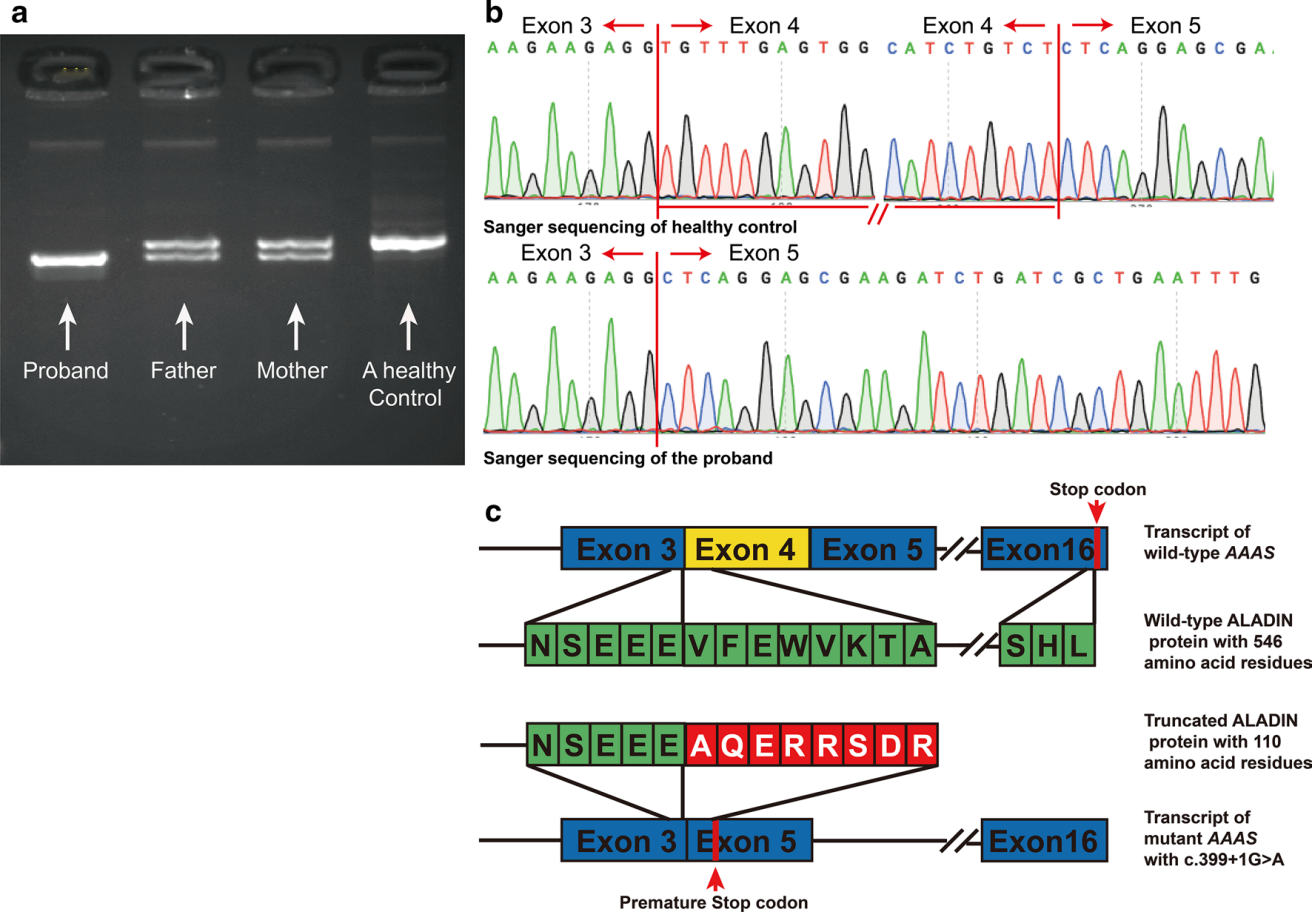
Transcripts of wild-type and mutant (c.399 + 1G > A) *AAAS*. **a** Agarose gel electrophoresis of the proband, the parents and healthy controls. **b** Sequencing of the purified band. The front band lacks exon 4. **c** Transcripts and translation of wild-type *AAAS* and mutant *AAAS*. The exon 4 skipping resulted in a premature stop codon and truncated ALADIN protein

Case 5 (Table 3) harboured a novel variant c.399 + 1G > A at the splicing juncture site at the end of exon 4 in the *AAAS* gene, which was predicted to affect splicing, as the wild-type motif “TCTgtaagt” was changed to “TCTataagt” without uncovering a cryptic splice site. Agarose electrophoresis of the cDNA from the proband, the parents and a healthy control demonstrated that the parents had two bands: one was a higher-molecular-weight band of the same size as that of the normal control individual, and the other was a lower-molecular-weight band of the same size as that of the patient. Sanger sequencing confirmed the exon 4 skipping in the lower-molecular-weight molecular band, and the protein was predicted to be truncated (Fig. 2).

## Discussion

In the present study, we recruited 70 infants and children with PAI and sequentially performed *CYP21A2* gene Sanger sequencing, MLPA testing and biochemical analysis plus detailed clinical examinations and found an overall 84.3% (59/70) diagnostic rate for CAH, with 91.5% (54/59) of CAH patients showing positive genetic findings. Excluding CAH, for uncharacterized PAI, we found that 72.7% (8/11) of the cases had positive genetic findings by WES and Array-CGH, which is lower than the rate reported in a previous review by Guran et al. (81%) [19, 29]. Including CAH, a total of 88.6% (62/70) of the children were determined to have a positive genetic test, which is lower than the 94.2% positivity rate detected by Perry et al. [2].

Among our 21-OHD CAH patients, the most prevalent *CYP21A2* variants were c.293-13C > G (31.4%), Del (18.6%), p.I173N (17.0%), E3 Δ8 (5.1%), p.R483PfsX58 (4.2%) and p.R357W (3.4%). The first three variants, namely, c.293-13C > G, Del and p.I173N, were consistent with the most frequent variants in the Asian population [30–34]; however, our data showed higher E3 Δ8 and p.R483PfsX58 variant prevalence rates and a lower p.R357W variant prevalence. Ethnicity-based differences were evident among different studies; the frequencies of p.P31L and p.V282L were 1.7% and 0.9% in our study, respectively, and while the p.V282L variant had a dramatically high frequency in Argentina and Brazil [35–37], the p.P31L variant was frequent in Serbia [38] (Table 1). Thus, the genetic background of 21-OHD differs by ethnicity.

Novel variants, namely, c.651 + 2T > G and c.833dupT, were found in the *CYP21A2* gene in two children (Table 2), and these variants were predicted to be damaging. Variant c.651 + 2T > G located at the end of exon 5 was predicted to alter the wild-type donor site and most likely lead to whole exon 5 skipping, which would result in the loss of 184–217 amino acid residues. With 9 highly conserved residues [39], these residues are essential to construct important functional domains, such as steroid-binding sites (residues 203–207) and large hydrophobic areas (residues 211–218). This splicing site variant likely impairs enzyme activity, because the proband displayed the SW phenotype. The other novel variant, c.833dupT, most likely causes complete loss of enzyme activity, because another comparable frameshift variant, c.923dupT (p.L308FfsX6), which retains more amino acid residues, causes complete loss of enzyme activity [40]. However, more functional studies are required to confirm the exact impact of the novel variants on enzyme activity in the future.

In our research, SW females had higher PGS scores than SV females, as reported by previous studies [41, 42], and the reason may be the higher 17-OHP and TES levels discovered in SW patients (Fig. 1). Interestingly, compared with males, females had higher serum 17-OHP and cortisol levels in both the SW and SV groups, which is consistent with another study [32]. Regarding the genotype–phenotype correlation, the PPV for group 0 was 100%, which is higher than those in most similar studies, while the PPVs in group A (82.6%) and group B (84.6%) were in accordance with those in previous studies [27, 32, 35, 37, 38]. Surprisingly, we found that 8.5% (5/59) of the patients clinically presented with the SW (n = 4) and NC (n = 1) phenotypes without biallelic variants (Additional file 6: Table S3). Studies have reported that monoallelic variants and absent variants accounted for 2.2% to 24% of enrolled children [27, 33, 36–38, 43]. The aetiology is unknown. We deduced that an amplification allele dropout effect may occur due to the high similarity between the *CYP21A1P* and *CYP21A2* genes. Additionally, the *CYP21A2* gene promoter region and other intronic variants that have not been analysed in studies may also reduce transcriptional activity [21].

In our uncharacterized PAI patients, we detected a total of 7 pathogenic variants, including one chromosome microdeletion and 5 novel variants in the *NR0B1*, *AAAS*, *NNT* and *ABCD1* genes. The *NR0B1* gene is the most frequent gene responsible for X-linked AHC in males (Table 4). The absence of *NR0B1* is proposed to cause progenitor cells to prematurely differentiate into steroidogenic cells without adequate maturity [44, 45]. We found two patients with one novel variant (c.323–324 CG > GA and c.1231_1234delCTCA) and one infant with an *NR0B1* deletion that manifested as PAI, mineralocorticoid deficiency and diminished adrenal glands; however, no hypogonadotropic hypogonadism was noted due to the patient’s young age. We also found two novel homozygous pathologic variants in the *AAAS* gene (c.399 + 1G > A and c.250delT) in 3 patients (Table 3, cases 6, 7, and 8) from 2 families, and both parents were consanguineous. Alacrima and AI were found in all three patients; however, achalasia was observed in only an 8-year-old boy, manifesting as swallowing difficulties. The other two sisters shared the same genotype with different phenotypes. These results suggest that careful estimation should be performed in every child with PAI with alacrima or achalasia. The *ABCD1* gene is another causative gene that is often observed in male patients and results in X‐linked ALD [45]. The clinical presentation of X‐linked ALD is variable, and no phenotype-genotype correlation has been observed [46]. These phenotypes include cerebral, adrenal, spinal cord and peripheral nerve involvement [46]. Fifty percent of affected children ultimately develop adrenomyeloneuropathy within 10 years [47]. Our patients showed clinical PAI, elevated serum long-chain fatty acid levels and normal neurological examination results but had white matter lesions detected by magnetic resonance imaging. We also found a novel pathogenic variant in the *NNT* gene (c.2274delT). Researchers reported that 53% of patients with *NNT* gene variants presented with hyperpigmentation, while 17% had mineralocorticoid deficiency [48]. Cardiac and thyroid involvement may also exist [49]. Therefore, a close long-term follow-up is still needed, as our patient presented with only hyperpigmentation. Evident genetic defects were not detected in three PAI patients, and we deduced that our current test methods may fail to identify deep intronic variants and epigenetic changes [17–20].

**Table 4:** Gene spectrum of uncharacterized PAI in other ethnic groups

Country	*STAR*	*NR0B1*	*SAMD9*	*AAAS*	*NNT*	*MC2R*	*CDKN1C*	*AIRE*	*CYP11A1*	*MRAP*	*NR5A1*	*ABCD1*	*CYP11B1*	Unknown	Positive percentage (%)
Japan [17]^a^	19	18	7	2	2	1	1	–	0	0	0	–	–	9	84.7
Canada [18]^a^	5	0	–	0	0	1	–	3	0	–	–	0	–	2	81.8
Turkey [19]^a+b^	11	12	0	1	7	25	0	0	9	9	1	2	0	18	81.0
UK Study [20]^c^	0	4	0	2	2	1	0	2	5	0	0	0	1	26	41.9
China (this study)^c+d^	0	3	0	3	1	0	0	0	0	0	0	1	0	3	72.7

^a^Targeted gene sequence

^b^Next-generation sequencing

^c^Whole-exome sequencing

^d^Array-based comparative genomic hybridization

## Conclusions

In conclusion, the genetic causes of PAI in children are very diverse, with a high prevalence of 21-OHD. A total of 7 novel gene variants were detected in the *CYP21A2*, *NR0B1, AAAS* and *NNT* genes in our cohort. SW 21-OHD females showed higher PGS scores than SV females, which was consistent with the higher levels of 17-OHP and TES. Ethnicity-based differences existed in the genetic variant spectrum of the *CYP21A2* gene. Evident genetic variants were not detected in 11.43% of the PAI patients despite our combined genetic testing protocol, suggesting that testing tools must be improved in future studies.

## Supplementary Information

**Additional file 1: Fig. S1.** Diagnostic algorithm for PAI patients. For a patient with primary adrenal insufficiency, a high serum level of 17-OHP suggests CAH, and he or she was subjected to CYP21A2 sequencing and MLPA. Children with normal or lower 17-OHP levels accompanied by developmental delay, intellectual disability or congenital anomalies underwent Array-GCH examination. WES was performed for the remaining patients.

**Additional file 2: Fig. S2.** Sanger sequencing of novel variants in the CYP21A2 gene. a c.833dupT. b c.651+2T>G.

**Additional file 3: Fig. S3.** Variants and Xp21.2 microdeletion in uncharacterized PAI. a c.1552C>T in the ABCD1 gene (case 1, NC_000023.1: g.153005609C>T; NM_000033.4; c.1552 C>T, reference protein NP_000024: p.R518W, rs128624224). b c.323-324CG>GA in the NR0B1 gene (case 3, NC_000023.1: g.30327156-30327157CG>GA; NM_000475.5, c.323-324 CG>GA, reference protein NP_000466: p.S108X). c c.1231_1234delCTCA in the NR0B1 gene (case 4, NC_000023.1: g.30322873-30322876delCTCA; NM_000475.5, c.1231_1234delCTCA, reference protein NP_000466: p.L411Vfs*6). d c.399+1G>A in the AAAS gene (case 5, NC_000012.1: g.53709118G>A; NM_015665.6, c.399+1G>A, reference protein NP_056480: p.?). e c.250delT in the AAAS gene (cases 6 and 7, and NC_000012.1: g.53714350delT; NM_015665.6: c.250delT, reference protein NP_056480: p. W84Gfs*10). f c.2274delT in the NNT gene (case 8, NC_000005.1: g.43656054delT; NM_182977.3, c.2274delT, reference protein NP_036475: p.I758Mfs*10). g 2.6M microdeletion on Xp21.2 (case 2). For Sanger sequencing, all sequences were forward sequences except for the patient in case 3 and his parents. The patient case 3 had a de novo variant, and his mother had the wild-type allele of the NR0B1 gene. Their sequences were reverse sequences.

**Additional file 4: Table S1.** PCR primers for CYP21A2 amplification.

**Additional file 5: Table S2.** PGS of CAH patients.

**Additional file 6: Table S3.** Variants in CYP21A2 gene.

**Additional file 7: Table S4.** Clinical features of CAH patients without pathologic biallelic variants.

## Data Availability

The datasets in the current study are available in the Mendeley repository (https://github.com/Alice-lzl-CZ/Public-data.git). Relevant genetic polymorphisms have been submitted to NCBI ClinVar (SCV001652698, SCV001652697, SCV001652696, SCV001652695, SCV001652694, SCV001623017). Transcripts data were downloaded from UCSC Genome Brower (http://genome.ucsc.edu/cgi-bin/hgGateway), accession number: NM_000500.9, NM_000033.4, NM_000475.5, NM_015665.6 and NM_182977.3.

## Abbreviations

PAI: Primary adrenal insufficiency
ACTH: Adrenocorticotrophic hormone
CAH: Congenital adrenal hyperplasia
PGS: Prader genital scale
17-OHP: 17-Hydroxyprogesterone
21-OHD: 21-Hydroxylase deficiency
Array-CGH: Array-based comparative genomic hybridization
WES: Whole-exome sequencing
TES: Testosterone
DHEA-S: Dehydroepiandrosterone sulphate
HSF: Human Splicing Finder
PPV: Positive predictive value
SW: Salt wasting
SV: Simple virilization
NC: Non-classic
MLPA: Multiplex ligation-dependent probe amplification
ALD: Adrenoleukodystrophy
X-linked AHC: X-linked adrenal hypoplasia congenita
AI: Adrenal insufficiency
IQR: Inter quartile range
