# Supplementary figures and images for: Genetic aetiology of primary adrenal insufficiency in Chinese children
Source: BMC Med Genomics. 2021 Jun 30;14:172. doi: 10.1186/s12920-021-01021-x (PMC8243448; doi:10.1186/s12920-021-01021-x)

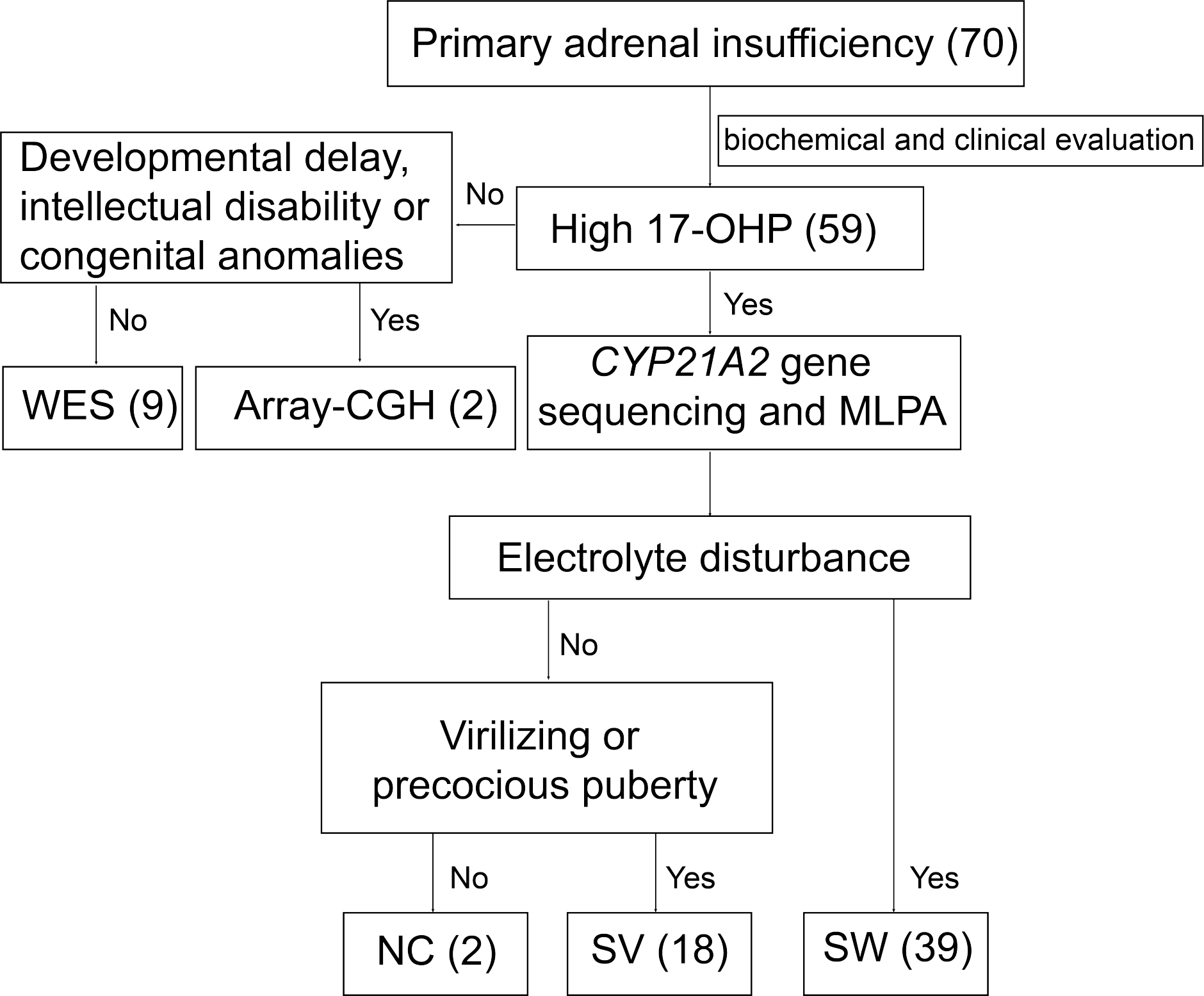

Supplement: Supplementary file 1 — Additional file 1: Fig. S1. Diagnostic algorithm for PAI patients. For a patient with primary adrenal insufficiency, a high serum level of 17-OHP suggests CAH, and he or she was subjected to CYP21A2 sequencing and MLPA. Children with normal or lower 17-OHP levels accompanied by developmental delay, intellectual disability or congenital anomalies underwent Array-GCH examination. WES was performed for the remaining patients. [file 12920_2021_1021_MOESM1_ESM.pdf]

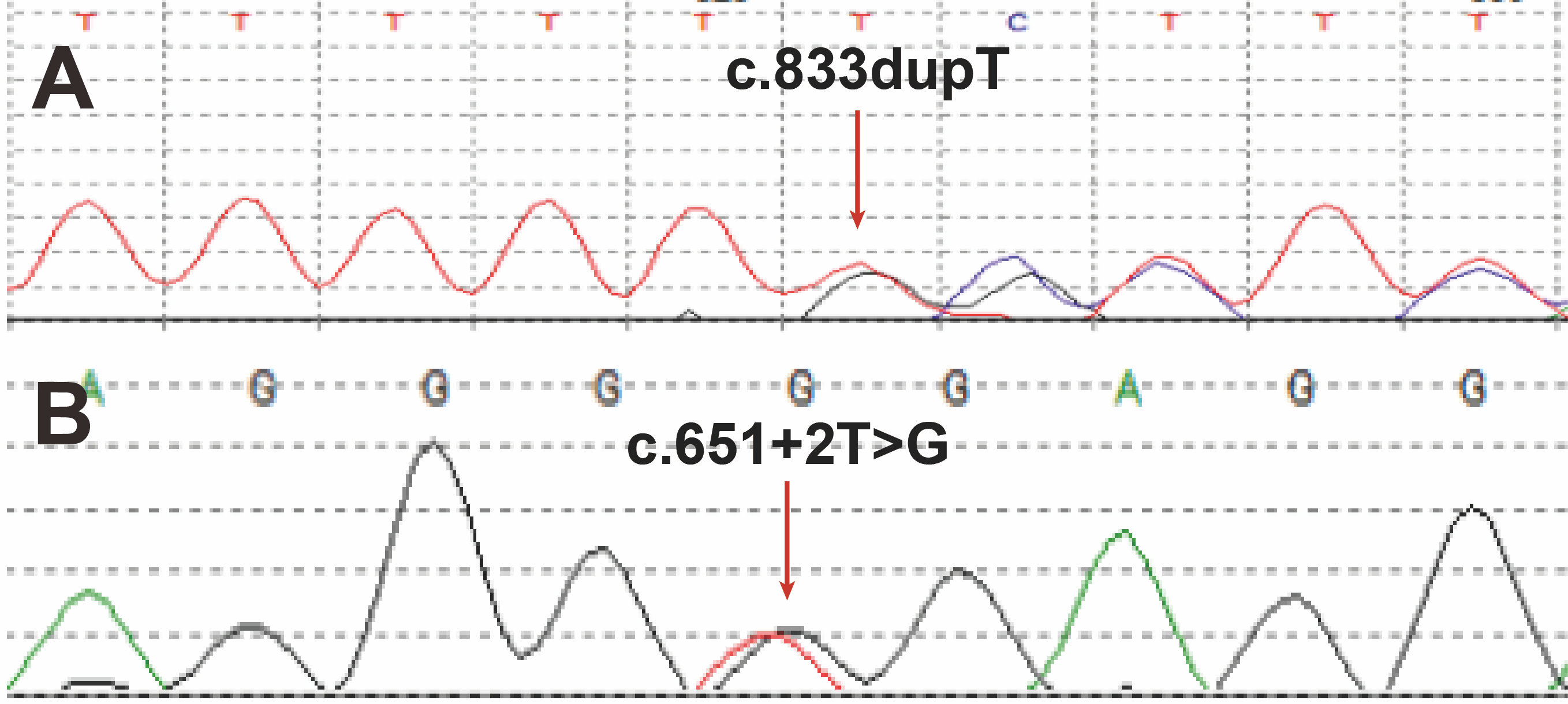

Supplement: Supplementary file 2 — Additional file 2: Fig. S2. Sanger sequencing of novel variants in the CYP21A2 gene. a c.833dupT. b c.651+2T>G. [file 12920_2021_1021_MOESM2_ESM.tif]

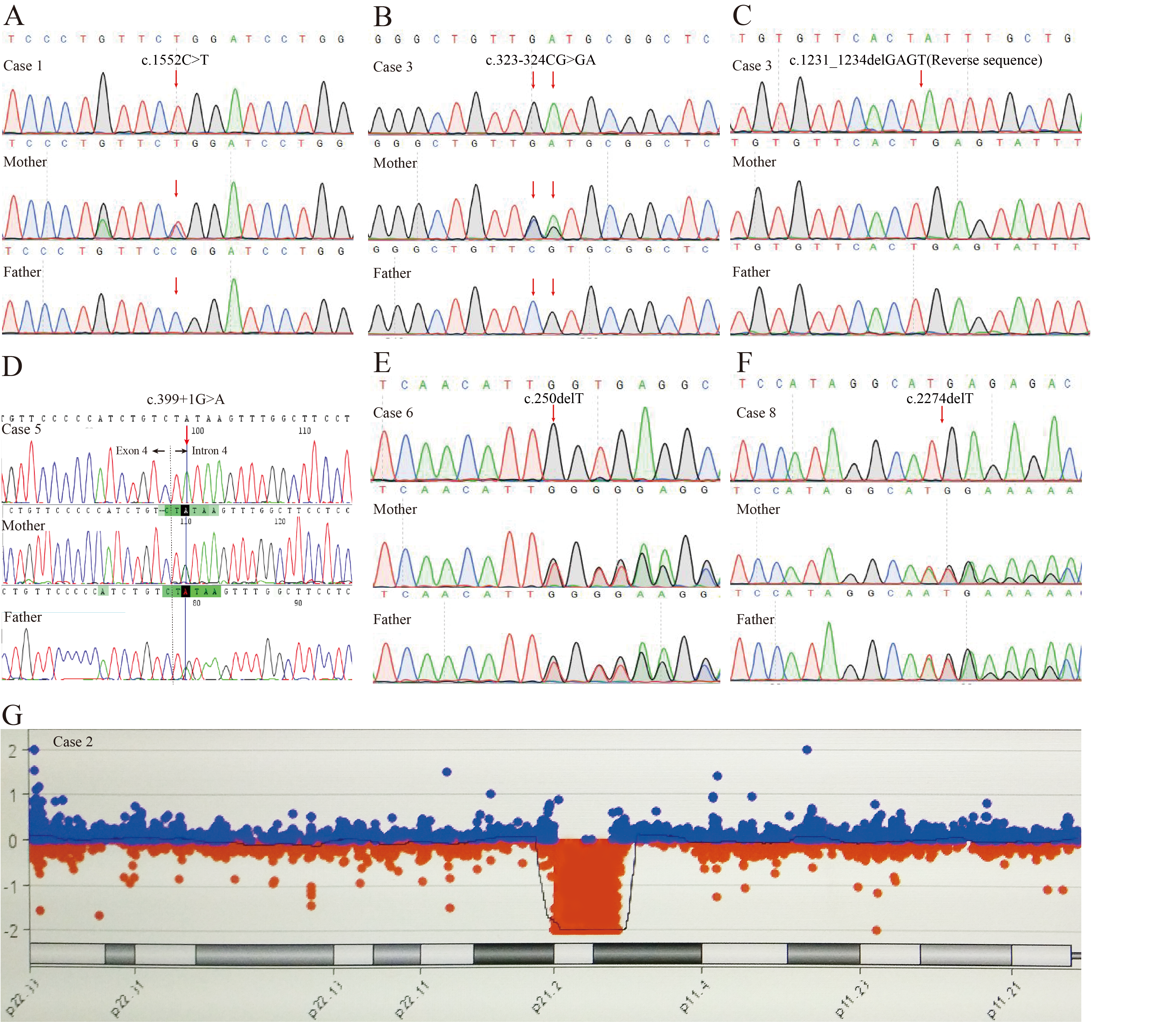

Supplement: Supplementary file 3 — Additional file 3: Fig. S3. Variants and Xp21.2 microdeletion in uncharacterized PAI. a c.1552C>T in the ABCD1 gene (case 1, NC_000023.1: g.153005609C>T; NM_000033.4; c.1552 C>T, reference protein NP_000024: p.R518W, rs128624224). b c.323-324CG>GA in the NR0B1 gene (case 3, NC_000023.1: g.30327156-30327157CG>GA; NM_000475.5, c.323-324 CG>GA, reference protein NP_000466: p.S108X). c c.1231_1234delCTCA in the NR0B1 gene (case 4, NC_000023.1: g.30322873-30322876delCTCA; NM_000475.5, c.1231_1234delCTCA, reference protein NP_000466: p.L411Vfs*6). d c.399+1G>A in the AAAS gene (case 5, NC_000012.1: g.53709118G>A; NM_015665.6, c.399+1G>A, reference protein NP_056480: p.?). e c.250delT in the AAAS gene (cases 6 and 7, and NC_000012.1: g.53714350delT; NM_015665.6: c.250delT, reference protein NP_056480: p. W84Gfs*10). f c.2274delT in the NNT gene (case 8, NC_000005.1: g.43656054delT; NM_182977.3, c.2274delT, reference protein NP_036475: p.I758Mfs*10). g 2.6M microdeletion on Xp21.2 (case 2). For Sanger sequencing, all sequences were forward sequences except for the patient in case 3 and his parents. The patient case 3 had a de novo variant, and his mother had the wild-type allele of the NR0B1 gene. Their sequences were reverse sequences. [file 12920_2021_1021_MOESM3_ESM.tif]
