# Supplementary material for: Genetic aetiology of primary adrenal insufficiency in Chinese children
Source: BMC Med Genomics. 2021 Jun 30;14:172. doi: 10.1186/s12920-021-01021-x (PMC8243448; doi:10.1186/s12920-021-01021-x)
Supplement: Supplementary file 4 — Additional file 4: Table S1. PCR primers for CYP21A2 amplification. [file 12920_2021_1021_MOESM4_ESM.docx]

**Table S1** **PCR primers for CYP21A2 amplification**

| **Primers for *CYP21A2*** | **Sequences** |
| --- | --- |
| Primer1F | 5’-AAACAGTCTACACAGCAGGAG-3’ |
| Primer1R | 5’-CTTGGAGCATGTAGTCCATCA-3’ |
| Primer2F | 5’- TGGAGGGACATGATGGACTA-3’ |
| Primer2R | 5’- GCTGGGTGAAATGGAACAATC-3’ |
