# Supplementary material for: Genetic aetiology of primary adrenal insufficiency in Chinese children
Source: BMC Med Genomics. 2021 Jun 30;14:172. doi: 10.1186/s12920-021-01021-x (PMC8243448; doi:10.1186/s12920-021-01021-x)
Supplement: Supplementary file 5 — Additional file 5: Table S2. PGS of CAH patients. [file 12920_2021_1021_MOESM5_ESM.docx]

| **Variable** | | **SW （N=39）** | | **SV （N=18）** | | **NC （N=2）** | |
| --- | --- | --- | --- | --- | --- | --- | --- |
|  |  | **Male (N=23)** | **Female (N=16)** | **Male (N=5)** | **Female (N=13)** | **Male (N=1)** | **Female (N=1)** |
| Initial age | | Neonate-5M | Neonate-3M | 1Y-6Y | Neonate-6Y | 4M | 7Y |
| PGS | Grade 0 | - | 0 | - | 0 | - | 1 |
|  | Grade 1 | - | 0 | - | 6 | - | 0 |
|  | Grade 2 | - | 1 | - | 2 | - | 0 |
|  | Grade 3 | - | 9 | - | 3 | - | 0 |
|  | Grade 4 | - | 5 | - | 2 | - | 0 |
|  | Grade 5 | - | 1 | - | 0 | - | 0 |
|  | Grade 6 | - | 0 | - | 0 | - | 0 |

**Table S2 PGS of CAH patients**

N: number; Y: years; M: months; SW: salt wasting; SV: simple virializing; NC: non-classic; PGS: Prader genital stage; M: months; Y: years.
