# Supplementary material for: Genetic aetiology of primary adrenal insufficiency in Chinese children
Source: BMC Med Genomics. 2021 Jun 30;14:172. doi: 10.1186/s12920-021-01021-x (PMC8243448; doi:10.1186/s12920-021-01021-x)
Supplement: Supplementary file 6 — Additional file 6: Table S3. Variants in CYP21A2 gene. [file 12920_2021_1021_MOESM6_ESM.docx]

**Table S3 Variants in *CYP21A2* gene**

| **Variants** | **dbSNP ID** | **HGMD** |
| --- | --- | --- |
| c.293-13C>G | rs6467 | [CS880069](http://grch37.ensembl.org/Homo_sapiens/Variation/Summary?db=core;g=ENSG00000231852;r=6:32006042-32009447;t=ENST00000418967;vf=681887292) |
| c.518T>A | rs6475 | CM880020 |
| c.332_339delGAGACTAC | rs387906510 | CD941657 |
| c.1451_1452delGGinsC | rs397509367 | CX920958 |
| c.1069C>T | rs7769409 | CM900080 |
| c.923dupT, | rs267606756 | CI920933 |
| c.92C>T | rs9378251 | CM994449 |
| c.955C>T | rs7755898 | CM880023 |
| c.[710T > A;713T > A;719T > A] | rs786204728 | PMID: 2845408 ^a^ |
| c.844G>T | rs6471 | CM880022 |
| c.1223-1G>A | rs151344503 | CM930185 |
| c.874G>A | rs201552310 | CM031955 |
| c.913G>A | rs151344505 | CM021255 |
| c.1451G>C | rs200005406 | CM031958 |

^a^ This pathogenic variant has no HGMD annotation but was proved to be pathogenic by *Higashi Y, Tanae A, Inoue H, Hiromasa T, Fujii-Kuriyama Y. Aberrant splicing and missense mutations cause steroid 21-hydroxylase [P-450(C21)] deficiency in humans: possible gene conversion products. Proc Natl Acad Sci U S A. 1988 Oct;85(20):7486-90.* PMID 2845408
