# Supplementary material for: Genetic aetiology of primary adrenal insufficiency in Chinese children
Source: BMC Med Genomics. 2021 Jun 30;14:172. doi: 10.1186/s12920-021-01021-x (PMC8243448; doi:10.1186/s12920-021-01021-x)
Supplement: Supplementary file 7 — Additional file 7: Table S4. Clinical features of CAH patients without pathologic biallelic variants. [file 12920_2021_1021_MOESM7_ESM.docx]

**Table S4 Clinical features of CAH patients without pathologic biallelic variants**

| **Probands** | **Gene variants** | **Stimulation tests (baseline or baseline/peak)** | | | | | **Electrolyte** | | | **Adrenal CT imaging** | **Main clinical presentation** |
| --- | --- | --- | --- | --- | --- | --- | --- | --- | --- | --- | --- |
|  |  | **ACTH**  **(pg/ml)** | **Cortisol**  **(baseline)**  **(ug/dl)** | **Cortisol**  **(peak)**  **(ug/dl)** | **17-OHP**  **(ng/ml)** | **TES**  **(ng/dl)** | **K^+^**  **（mmol/l）** | **Na^+^**  **（mmol/l）** | |  |  |
| Case 1 | c.913G>A/- | 27 | 5.82 | 41.9 | 85.6 | 192.09 | 5.0 | 140 | Bilateral adrenal enlargement | | NC: pigmentation |
| Case 2 | c.293-13c>G/- | >1250 | 11.29 | ND | 951.75 | 1600.00 | 5.8 | 118 | ND | | SW: pigmentation,  growth restriction, hypothyroidism, virializing |
| Case 3 | R483PfxX58/- | 57.3 | 4.24 | ND | 267.31 | 186.70 | 5.6 | 109 | Bilateral adrenal enlargement | | SW: pigmentation,  growth restriction,  virializing |
| Case 4 | -/- | 29.5 | 6.54 | 12.46 | 196.42 | 32.13 | 5.6 | 113 | Bilateral adrenal enlargement | | SW: pigmentation,  vomiting |
| Case 5 | -/- | 113.0 | 5.35 | ND | 53.33 | 157.66 | 5.7 | 134 | Bilateral adrenal enlargement | | SW: pigmentation,  early birth, vomiting |

*NC* non-classic, *SW* salt wasting, *SV* simple virializing, *ND* not detected, *17-OHP* 17-hydroxyprogesterone, *ACTH* adrenocorticotrophic hormone, *TES* testosterone, *DHEAS* dehydroepiandrosterone. Normal ranges ACTH <46 pg/ml, cortisol 5-25 ug/dl, 17-OHP 1 month – 1 year 1.06-40.41 ng/ml, 1 year-13 years 0.07-1.53 ng/ml, TES female 0-31 ng/dl, male 0-6 years 3-32 ng/dl, 7-12 years 3-68 ng/dl, DHEAS 35-430 ug/dl.
